# Obtaining a second opinion is a neglected source of health care inequalities

**DOI:** 10.1186/s13584-019-0289-5

**Published:** 2019-01-16

**Authors:** Jochanan Benbassat

**Affiliations:** Myers-JDC-Brookdale Institute, Department of Health Policy Research, PO Box 3886, 91037 Jerusalem, Israel

**Keywords:** Second opinion, Health seeking behaviors, Health policy, Doctor-patient communication

## Abstract

Observational studies have detected discrepancies between two expert interpreters of imaging and histopathological studies. Furthermore, in a substantial proportion of patients, an independent second opinion disagreed with the first one. Therefore, it is widely accepted that patients have a right to obtain a second opinion and, in case of divergent opinions, to deliberate and choose the option that they believe is most consistent with their individual circumstances. However, doctors are less likely to inform old and poorly educated patients about the possibility of seeking a second opinion, and this may contribute to healthcare inequalities. Hence the importance of (a) promoting doctors’ self-awareness of a possible tendency to discriminate against old and poorly educated patients, and (b) creating programs within the healthcare system that would help patients in seeking a second opinion, suggest specialists for the specific problem of the patient, and provide tools to reconcile between discrepant opinions.

Yerushalmy [1] is credited for being the first to report that a competent radiologist misses as many as 32% of the lesions on a single chest x-ray reading and disagrees with himself in about one-fifth of two readings of the same x-ray. Since then, there have been repeated reports of discrepancies in the interpretations of imaging and histopathological studies, as well as between clinical assessments. As late as 2015–2018, discrepancies between two expert interpreters have been reported in 22–57% of imaging studies [2–10] and in 25–37% of histopathology studies [11–14]. Discrepancies between clinical assessments have been reported in 20% of the cases of breast cancer [15], in 35% of patients in whom spinal surgery was recommended [16], and in 20 to 38% of patients with pancreatic cancer [17].

Therefore, Yerushalmy’s recommendation in the 1950s that dual reading may contribute to radiography is appropriate also for the 2010s and not only for radiography. Today, it is widely agreed that, unless it may delay a life-saving intervention, patients have a right to an independent second opinion [18], and that second opinions may lower health care costs while reducing both over-and under-treatment [19]. Several authors have recommended creating programs within the healthcare system that would help patients in seeking a second opinion, suggest specialists for the specific problem of the patient, and provide tools to reconcile discrepant opinions [20]. However, as of now such programs are rare, and obtaining a second opinion is mostly initiated by patients.

In their 2017 paper in the IJHPR, Shmueli et al. [21] join the recommendation to encourage patients to seek a second opinion. The authors surveyed a representative sample of the Israeli population and found that 41% had sought a second opinion because of doubts about diagnosis or treatment (38%), search for a sub-specialty expert (19%) and dissatisfaction with the first opinion (19%). As many as 56% reported a difference between the two opinions and 91% of them preferred the second.

These findings are consistent with those reported by others. Systematic reviews of the literature have indicated that the quest for a second opinion in different patient populations varied widely between 7 and 36% [20] and between 1 and 88% [22]. Patients sought a second opinion in order to confirm a diagnosis or treatment, or obtain information about persistent symptoms or treatment complications [22–24]. Systematic reviews have also indicated that the second opinion confirmed the original diagnosis or treatment in 43–82% of cases [20], and yielded a change in the diagnosis, treatment, or prognosis in 12–69% [20], 10–62% [23] and 2–51% [22]. Of particular interest were the outcomes of a program (Best Doctors, Inc.) that allows employee-beneficiaries to request free second opinion and to have trained physicians summarize the cases, identify unresolved clinical questions, and forward the cases to specialists for independent assessments and recommendations. It was found that a second opinion resulted in changes in diagnosis (15%), treatment (37%), or both (11%). The clinical impact of a second opinion was estimated as moderate / major in 21% of cases for diagnosis and 31% of cases for treatment. Most patients (95%) were satisfied with the experience, but fewer (61%) planned to follow the recommendations [24].

In summary, the main finding of these surveys was that a second opinion disagreed with the first one in a substantial proportion of patients [20–23]. The main limitation of these surveys is the absence of a gold standard that would identify “correct” opinions. Still, it is widely agreed that patients have a right to an independent second opinion and, in case of divergent opinions, *to deliberate and choose the option that they believe is most consistent with their individual preferences.*

Where should we go from here? I believe that further surveys aimed at determining the proportion of patients seeking a second opinion and their reasons for doing that are not warranted. However, the findings that patients with lower socioeconomic status and education were less likely to seek a second opinion [22, 25, 26] and that physicians were more likely to inform young and educated patients about the possibility of seeking it [27] are highly disturbing. These findings identify an additional source of health care inequalities.

One could envisage administrative interventions that would reduce these inequalities. For example, the ministry of health or individual health plans could include the procurement of a second opinion into the charter of patients’ rights and prominently display these rights in outpatient facilities. The ministry of health may assign to family doctors the responsibility for encouraging patients with chronic disorders, cancer and those who consider surgical or risky diagnostic / treatment intervention to seek a second opinion. Finally, health plans may disseminate the information that differences of opinion are common and provide instructions that would help both patients and their family doctors in finding specialists for specific problems. Still, I feel that administrative interventions will be only partially effective if not supplemented by doctors’ awareness and cooperation.

Some doctors admit having negative feelings about certain patients. However, only few are aware that these feelings may lead to a subconscious discrimination against elderly [28] and poor [29] patients. Doctors should be reminded of the undisputed association between all-cause mortality and *socioeconomic status* (income, education) [30, 31]. In other words, poor, uneducated and older patients are *more* susceptible to disease. *Any symptom or sign in a poor, elderly, or uneducated person may herald a more serious disease than in patients without these risk indicators,* just as the probability of a life-threatening infection in a neutropenic patient with fever is higher than that in a non-neutropenic person with the same degree of fever. Hopefully, doctors’ awareness that poverty, lower education and old age are risk indicators for disease will reduce their subconscious discrimination against such patients.

Second, doctors should be aware of the main barriers that prevent patients from seeking a second opinion. Focus groups have indicated that these barriers are patients’ sense of shock, pressure of time, information overload, and fear of jeopardizing the patient-physician relationship [32]. Therefore, an appropriate delivery of “bad news” would include an *unhurried* consultation, patient’s *encouragement* to seek a second opinion, and *scheduling a follow-up visit in order to respond to additional patient’s questions, provide additional information and gain an insight into the patient’s understanding of his / her disease*.

Third, doctors should help patients cope with divergent first and second opinions. Evidence suggests that a major drive to seek a second opinion is patient’s dissatisfaction with the first one. In-depth patients’ interviews indicated that they *wanted the consultant to apply his/her knowledge to the specifics of their individual cases, and were disappointed and distrustful when physicians cited only general prognostic statistics* [33]. Both family doctors and consultants can gain an insight into the specifics of the patient’s individual case by asking questions such as *“it would help me advise you if you told me what you think about your disease”* or *“what worries you most about your disease”* or *“what do you want most to avoid”* or *“what do you expect from the treatment”.*
